# Rapid Bioethical Assessment for *Mycobacterium tuberculosis* and Host Genetic Study in Amhara Regional State, Ethiopia: Towards a Context-Specific Ethical Approach

**DOI:** 10.4314/ejhs.v33i3.4

**Published:** 2023-05

**Authors:** Daniel Mekonnen, Abaineh Munshea, Endalkachew Nibret, Awoke Derbie, Mastewal Wubetu, Mengistie Taye, Taye Zeru, Belay Bezabih, Muluken Azage, Kidist Bobosha, Abraham Aseffa

**Affiliations:** 1 Department of Medical Laboratory Sciences, School of Health Science, College of Medicine and Health Sciences, Bahir Dar University, Ethiopia; 2 Health Biotechnology Division, Institute of Biotechnology, Bahir Dar University, Ethiopia; 3 Department of Biology, Bahir Dar University, Ethiopia; 4 The Centre for Innovative Drug Development and Therapeutic Trials for Africa (CDT-Africa), Addis Ababa University, Ethiopia; 5 Department of Ethiopia Langue(s) and Literature-Amharic, Faculty of Humanities, Bahir Dar University, Ethiopia; 6 Department of Animal Science, College of Agriculture and Environmental Sciences, Bahir Dar University, Ethiopia; 7 Amhara Public Health Institute, Ethiopia; 8 Department of Environmental Health, School of public health, College of Medicine and Health Sciences, Bahir Dar University, Ethiopia; 9 Armauer Hansen Research Institute, Ethiopia

**Keywords:** Rapid ethical assessment, tuberculosis, genomics, Consent, Ethiopia

## Abstract

**Background:**

Rapid Ethical Assessment (REA) is a rapid qualitative study anticipated to understand the ethical sphere of the research setting prior to recruiting study subjects. This study assessed the communities' knowledge about tuberculosis (TB) and research, understand the social arrangements advisable for recruiting research participant and appraised the information provision and consent process.

**Methods:**

The study was conducted in Amhara region, Ethiopia from 5^th^-30^th^ January 2021. Google-based survey, face-to-face in-depth interview and focus group discussion were carried out to collect the data from researchers, data collectors, health professionals, TB program officers. A structured questionnaire was administered to assess the knowledge of TB patients and healthy controls about TB, research, gene, (co)evolution and consent process.

**Results:**

Over 71% of researchers were not satisfied with the current consent process, and 82.7% of researchers agreed that the best interest of the research participants was not adequately addressed in the current research practices in ANRS. TB patients and healthy controls misunderstood research and its goals. Participants advised the researchers to approach the community with the assistance of health extension workers (HEW) or religious/local leaders. Combined use of verbal and written based information provision at individual participant level is the preferred way for information provision.

**Conclusions:**

The adherence of researchers to standard information provision and consent process was very low. Healthy controls and TB patients have low level of knowledge and awareness about research, ethics and genomic research-related common terms. Hence, public education is required to strengthen the research ethics in the region.

## Introduction

History witnessed the unethical research practices and scandals such as the Tuskegee syphilis experiment, which followed patients between 1930 and 1972 by withholding available treatments (1–3), the Jewish chronic disease hospital study in which chronically ill patients were injected with live cancer cells to see if the cancer cells would be rejected(4–6) and the Willow Brook State School in New York in which mentally handicapped children were injected with viable hepatitis virus to study the course of infection (7, 8). Questionable research practices were also carried out in Africa during the colonial period (9). Unethical research practices still continue throughout the world (10). For instance, in Japan alone over 158 unethical studies were identified (11). South Africa San indigenous community who are African foci of genome research exposed to several unethical research (12). Ethiopia as one of the global foci of human and pathogen genomic studies (13), the knowledge of the society about genomic research and their social arrangement must be well documented.

The global research and human right community developed research ethics guidelines and declarations. Some of these include the 1947 Nuremberg Code which contains ten permissible experiments (14), the declaration of Helsinki (15), the 1982 Council for International Organizations of Medical Sciences guidelines (16), the 1996 international good clinical and laboratory practice guidelines (17, 18) and the International Conference on Harmonization Good Clinical Practice standards (19). These documents included the universal declarations on bioethics and human rights (20) and the universal declarations on the human genome and human rights (21). According to these guidelines, independent institutional committees must approve all research protocols in advance.

In Ethiopia, Research Ethics Review Committee were established at the University level in the 1970s and research ethics review guidelines were first developed in 1995 by the Ethiopian Science and Technology Commission (ESTC); the current Ministry of education. According to this national research ethics guideline, for any medical research all of the following eight criteria must be fulfilled: (1) The research must have ethical justification and scientific validity, (2) the research must have science and social value, (3) favorable risk-benefit ratio to research participants and their communities must be waited, (4) selection and enrollment of subjects must be fair, (5) privacy and confidentiality must be maintained, (6) the protocol must be reviewed by independent institutional review committee (7) written informed consent must be obtained from each participant and (8) the study must involve the community in decision making process about the design and conduct of the study (22).

In developing countries, obtaining consent is challenging due to several barriers such as illiteracy and limited understanding of the scientific rationale for the studies and lack of trust and confidence on research (23). Moreover, lack of proper training and resources dampen the promotion of high ethical standards by ethical review boards (24).

Rapid ethical assessment (REA) is often employed to understand and address context and disease specific ethical issues. In this regard, RE A is a qualitative study that is designed to map the ethical ground of the research setting prior to recruitment of study participants (25). Rapid ethical assessment mainly focuses on issues related to information provision and consent processes. Information provision and consent processes encompass the design of consent processes, community engagement exercises, training processes for pollsters, and the multiple activities before and during data collection (26).

Bioethics has neglected the topic of infectious disease (27). However, whole genome sequencing of pathogens and human is now expanding in unprecedented speed in various settings (13, 28, 29). Currently, ‘pathogenagnostic' approaches is used to isolate disease causing pathogens including novel pathogens from a clinical samples (30). This technology generates large and fine scale data which has several ethical issues over data sharing and interpretation (31, 32). Additionally, the use of these fine scale information to map chains of transmission in a certain geographic region may lead to discrimination (30–32).

Tuberculosis primarily affects the poor raises issues of social justice (33). Tuberculosis is a disease of poverty (34) and pathological intersection of political, economic, and biological processes (35). East Africa is the evolutionary foci of human and *M. tuberculosis (36)*, making it an ideal regions for (co)-evolutionary genomics study. Towards this, the research ethics process must be well documented and communities must be aware of the research processes. Thus, this study assessed the knowledge of the community about TB, research and terms such as genomics, genome, evolution and coevolution and further understand the social structure and appraised the information provision and consent process.

## Methods

**Study design, period and setting:** An ethnographic survey was carried out in ANRS, Ethiopia. The study was conducted from 5th to 30th January 2021. The perception of researchers' and institutional review board (IRB) members about REA was obtained using Google form. The Google form survey link was dispatched via email, Facebook and participants were reminded for five times to fill out the questionnaire. The qualitative views of participants about the social organization of Amhara community, knowledge of the general public about TB and research, the practices of researchers in the information provision and consent process were obtained through in depth interviews (IDIs) and focus group discussions (FGDs).

**Study population**: Researchers and IRB members were the study population in the online based survey. Researchers, IRB members, TB program officers, health professionals working in the directly observed treatment, short-course (DOTS) clinic, data collectors such as HEW, community members without TB and TB patients with age greater than 18 years participated in the IDI and FGD.

**Sampling methods and sample size**: Convenient sampling technique was applied to enroll TB patients and healthy controls. Purposive sampling method was used to recruit researchers, IRB members, data collectors, health professionals and leaders. The criteria for inclusion were experience on research and data collection. A total of 74 researchers and IRB members were invited to participate in the online survey. Of these, 52 (70.3%) fill out the survey questionnaire. A total of 21 IDIs and FGDs were carried out, which altogether enrolled 36 participants. Each IDI and FGD was records and coded with a combination of words and a number. For instance “REA-R01” means; the audio recorded for REA study from the researcher (R). The number “01” is the sequence of the record from each population category (Table 1).

**Table 1:** Summary of participants' category, interviews and ID of audio data collected in ANRS, January 2021

Participants' category	#IDI	# FGD	#participants in FGD	Audio ID
HEW and data collectors	1	1	5	REA-DC
TB program officers	-	1	7	REA-PO
TB clinic physicians	3	-	-	REA-HP
Community representatives	2	-	-	REA-CR
TB patients and health controls	5	-	-	REA-HC, REA-TB
Researchers	5	2	2+3=5	REA-R
IRB members	5	-	-	REA-IRB
Total	21	4	17	

**ID:** Identification code, **HEW:** Health Extension workers, **TB:** Tuberculosis, **IDI:** In-depth interview, **FGD:** Focus Group Discussion, **IRB:** Institutional Review Board

**Data collection**: Questions used for gathering the perceptions of researchers about REA were adopted from previous study (25). The IDI and FGD checklists were adopted from a study done in Gambia (26). The checklist was classified in to seven themes. These include (1) knowledge of participants about TB, (2) knowledge of participants about research, (3) approaching the community, (4) information provision process, (5) consent process, (6) blood sample collection and (7) genomic incidental finding (GIFs) and incentives (37, 38). For clarity purpose, edited transcription method was used to transcribe the audio data.

**Ethical consideration:** The study was approved by IRB of Science College of Bahir Dar University (IRB-SCBDU) with reference number PGRCSV/111/2012 and support letter was obtained from Amhara Public Health Institute (APHI). The study participants were informed about the purpose of the study, the importance of their participation in the study and their rights. Following information provision, oral consent was obtained since they were very aware of the research or the research was none invasive.

**Data quality control:** The questionnaires (25) and the check list (26) adopted from previous studies reevaluated for completeness before administering to participants. The transcribed information was evaluated against the audio by two researchers independently (AM, EN) to maintain descriptive validity. Additionally, some participants review the final transcribed information for consistency.

## Results

**Perceptions about REA**: Perceptions about REA was collected from 52 researchers and IRB officers, of which 46 (86.5%) were males. Of all participants, 37 (75.2%) had research ethics training. Table 2 summarizes the responses.

**Table 2:** Perceptions of researchers and IRB members, regarding REA in Amhara Regional State, January 2021 (N=52)

The yes/no questionnaire	Response N (%)
Do you think all participants understand consent forms well?	
Yes	10 (19.2)
No	42 (80.8)
Based on your experiences, are you satisfied with the way the consent process was designed and conducted?	
Yes	15 (28.8)
No	37 (71.2)
Do you think that the best interest of study participants is taken into consideration and adequately addressed through the current ethical appraisal and consent processes?	
Yes	9 (17.3)
No	43 (82.7)
Based on your experiences, what do you think are the most common problems in research consent process?	
Inadequate information	39 (75)
Lack of clarity	33 (63.5)
Language	27 (51.9)
Cultural difference	27 (51.9)
Undue expectations	14 (26.9)
Power imbalance	11 (21.2)
Coercion	5 (9.6)
Others	5 (9.6)
Do you think it is important to contextualize consent forms and consent processes to local settings?	
Yes	52 (100)
No	0 (0)
Do you agree with the idea of the study participant be approached in advance before the start of the study to get input for the development of the consent form and to find out how it should be administered?	
Yes	45 (86.5)
No	7 (13.5)
Do you think that study participants should be involved, in the development of consent forms and designing of the consent process so as to make it culture and setting sensitive?	
Yes	39 (75)
No	13 (25)
In your opinion, would REA serve adequately addressing the consent process issues and in making sure that ethical issues are very well addressed in a research process?	
Yes	37 (71.2)
No	15 (28.8)
From your experiences, has there been any initiative so far that involved study participants in the development and design of consent information sheet and consent process?	
Yes	6 (11.5)
No	46 (88.5)

**REA:** Rapid Ethical assessment, **IRB:** Institutional review board, **N:** Frequency

## Qualitative Views About REA

**Theme I: Knowledge of the community about tuberculosis:** Tuberculosis is known by the community as *“disease of cough”* (REA-PO01). *“TB is a transmissible disease and the cause is bad air”* (REA-TB02, REA-HC03). All TB patients and HC said that TB is not a heritable disease. This is because *“in our village, it is not limited to a particular family”.*

**A DOTS clinician classified TB patients into rural and urban**:

*“Rural communities are happy when they are diagnosed with TB. This is because, they have several experiences with TB patients who became cured after taking anti-TB drugs. On the contrary, people from the urban area become disappointed when they were diagnosed with TB. This is because they consider TB as disease of poverty and low socio-economic classes” (REA-HP02).*

The following is the story and knowledge of one TB-HIV co-infected patient about TB:

*“I was diagnosed with TB 10 years ago and cured. Then, I went into a remote commercial center for private work. There, I became sick and visited several health facilities. When my health condition became deteriorate, I left my work, returned to my family and then visited this hospital. Here, I diagnosed with TB lymphadenitis. I know that, TB can be converted to HIV. I thought that my TB is due to bad air and dirty blood. TB is not a heritable disease” (REA-TB01).*

**Theme II: Knowledge of community about research and the challenges:**

The majority of the community members do not understand the term “research” and its scope. To our surprise, the research literacy of the majority of middle level health professionals was low.

A 35 years old researcher said that:

*“I don't think that majority of the community can clearly define what does research mean. People often cannot differentiate research from medical service and supportive supervision”* (REA-R01). Similarly, one of the health professionals in the IDI said that “*the community views research as a medical service”* (REA-HP06). A young male TB patient said *“I do not know the difference between research and medical service. This is because I am from the rural area and I did not obtain any training before”* (REA-TB03).

The common challenges related to research in Amhara region includes the following: (1) *“some consider research as a means of incentive”* (REA-PO01), (2) *“others consider it as systematic means of harming the community”* (REA-PO01), *(3) “some others considered research as a secret business of groups and had nothing to do with the participants or to the community”* (REA-HP04) *and (4) “some viewed research as a mere academic duty of medical students”* (REA-HP04). *“The issue of social desirability bias is also a serious and hidden challenge”* (REA-R04).

**Theme III: Approaching the community**

In ANRS, there are different types of community structures (religious, political and social). To conduct community based study, one or more of these structures can be followed. The field data collector with 15 years of experience shared his experience:

*“During our work in Zarima, north Gondar, one police was spreading misinformation and campaigning against the research team. However, since the kebele leader and militias were with us, he was detained and advised. Hence, I advised researchers to include kebele leaders or militias or HEWs” (REA-HP04).*

**Figure 1:**
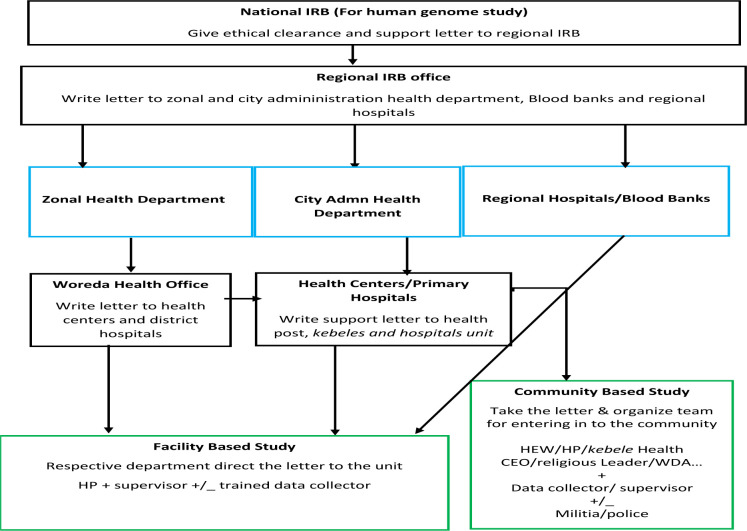
The research administrative structure in Amhara Regional State, 2021 ***IRB:*** Institutional review board, ***HEW:*** Health extension worker, ***HP:*** Health professional, ***WDA:*** Women development army, ***CEO:*** Chief Executive officer

In a FGD of three participants, *said that “Unless you have an arbitrator, people will not tell you the truth” (REA-R05).* In apparent contradiction to the above view, a none-TB control participants forwarded a different view; *“I believe that approaching the community with the kebele leaders might make some people unhappy due to some form of conflict of interests” (REA-HC04).* Taken together, different types of conflict of interests and conspiracy theories makes research and field works challenging in ANRS. For instance, one Ph.D student was killed in West Gojam Zone and two vehicles of Armaur Hansen Research Institute were burned out while collecting research data. Thus, the participants advised researchers to follow local regulations and recommendations outlined below (Figure 1).

**Theme IV: Information provision:**

“*Verbal-based information provision at individual level is preferable*” (REA-HP01). Another DOTS clinic physician said that *“both verbal and written based information is advisable and we usually use both during community health education”* (REA-HP06). *“Giving the information sheet can be considered as a sign of respect”* (REA-R05).

Almost all participants agreed that the community was unable to understand common terms related to TB genetic study such as “genetic”, “genome”, “DNA”, “evolution” and “co-evolution” (REA-PO01). In one FGD of researchers, they pointed out critical points which must be emphasized. “*During information provision, interpretation of words has a significant impact on participation or refusal. A standardized Amharic version might be desirable for providing balanced information” (REA-R04)*.

**Theme V: Consent process**

All participants said that, consent must be obtained at individual level. “*However, signature based consent not only leads to refusal but also likely to be biased. As a remedy, data collectors must be compassionate and friendly. Additionally, the researcher must explain clearly and adequately about the confidentiality of the data”* (REA-R05). Contrary to the above perceptions, one of the HC said that, *“asking a signature might be considered as a sign of respect”* (REAHC-04). *“When you provide adequate and clear information about the study and their rights, some participants even thank us for being part of the study”* (REA-PO01, REA-HP06).

**Theme VI: Blood sample collection**

Obtaining clinical samples from study participants always bearing a challenge. The rural and urban communities have distinctive explanation about blood sample.

“*Rural community believe that they don't have enough blood to give for research and even for life saving transfusion”* ((REA-HP02, REA-R01). “*The urban community fear for being traced by their blood”* (REA-R03). “*They are also suspicious about the sterility of the syringe and possible nerve damage”* (REA-R05).

Explaining the amount of blood required for the research is also challenging. As such, the study explored an alternative volume measuring device that is simple and easily understood by the local community. The recommendations are to inform the participant:

“*By using small syrup measuring cap ”, “to recall their previous experience if they gave blood for diagnostic purpose”, “by marking the test tube* or *the syringe*” and “*by using common terms like two to three saliva or tear drops”*.

**Theme VII: Genomic incidental findings and i ncentives**

Questions related to genomic incidental findings and research incentive were forwarded to IRB members only. In an Ethiopian context, human genomic study is ethically reviewed at the national level by Ministry of Education. Based on IDI with IRB members. *“Since regional state and most University IRB are level B, most of the recommendations for genomic study and the reporting of genomic incidental findings are not known by level B IRBs”* (REA-IRB01).

Research incentive is the source of disputes. *“As indicated in Amhara Regional IRB guideline, payment is made to participants if they spend over one and half hour within the research, have travel associated expense, and food is served*” (REA-IRB03).

## Discussion

This study assessed the knowledge of the community about “research”. The most of the general community have a low level of knowledge about “research” and “research ethics process”.

The major share of problems related to research ethics goes to the researchers. The prime goal of context and disease specific consent tools is to provide adequate, and clear information with their local language so as to help participants make informed decisions. Hence, regulatory bodies must give emphasis on whether the best interest of participants are maintained and the consent tool is appropriate to the study setting. Unfortunately, some researchers considered ethical review process as an additional unnecessary bureaucratic procedure and a mere administrative matter (25). Addissie *et al* (25) assessed the perceived relevance of introducing REA as a mainstream tool in Ethiopia.

Information is power for an informed decision. Hence, all components of the information sheet must be explained adequately and sufficiently with clear terms. The researcher must give enough time for each participant. Additionally, researchers and data collectors must know that obtaining consent need artistic and genuine approach. Researchers and data collectors must be compassionate, respectful and friendly when approaching participants. In addition to verbal based information, data collectors shall give written signed information sheet with full contact address. This will increase the confidence of participants and be taken as a sign of respect.

Written consent might lead to refusal or social desirability bias. Hence, a systematic effort has to be put to build trust. Researchers must be aware for possible conspiracy theories and misinformation against them during the courses of the study. Participants must be informed about the accountability of data collectors. They must be informed of the presence of local and international laws that prohibit the use of clinical samples and information other than purposes mentioned in the information sheet.

Being an ethnographic research, this study claims validity as a strength due to the fact that the researchers and participants are part of the setting. The limitations of the study might be the stringency and generalizability of the ideas and opinions due to lack of controls common to other types of research. Additionally, TB patients and health controls were not stratified by socio demographic, economic and educational factors. Furthermore, FGD was not carried out among DOTS physicians. Considerations of such factors in the future might help to obtain the more realistic view of the community about each theme.

In conclusion, majority of participants agreed that the general public do not know much about research, its goal and its difference with medical services. Additionally, not only the English names, but also the local Amharic language translations of terms such as genome, DNA, evolution and co-evolution are argot.

As a closing remark, the research team want to remind one of the popular proverbs in research ethics: *“Doing the right things does not bring success automatically, but compromising ethics almost always leads to failure”.* Hence, research should not be done at the cost of ethics and we must be ethical.
